# Conceptualising the impact of telehealth on rural workforce sustainability using system dynamics and the Gartner Hype Cycle

**DOI:** 10.1038/s44401-025-00044-1

**Published:** 2025-10-29

**Authors:** Sagda Osman, Kate Churruca, Mohammad S. Jalali, Louise A. Ellis, Jeffrey Braithwaite

**Affiliations:** 1 Centre for Healthcare Resilience and Implementation Science, Australian Institute of Health Innovation, Macquarie University, North Ryde, NSW Australia; 2 MGH Institute for Technology Assessment, Harvard Medical School, Boston, MA USA

**Keywords:** Health policy, Operational research

## Abstract

Clinician-to-clinician emergency telehealth addresses healthcare access barriers in Australia, yet its impact on workforce sustainability remains poorly understood. This study operationalises the Gartner Hype Cycle through system dynamics modelling to examine telehealth’s impact on rural workforce recruitment and retention. Grounded in stakeholder interviews, expert workshops, and published literature, we developed a system dynamics model capturing delays between recognising benefits and emerging limitations, incorporating three mechanisms through which telehealth affects rural on-site medical position attractiveness: tacit knowledge sharing, medicolegal implications, and clinical support access. Testing 75 parameter scenarios revealed only 15% maintained sustainable on-site staffing long-term. These scenarios involved conservative initial telehealth allocation, realistic expectation management, and scale-specific strategies recognising smaller facilities’ vulnerability. Varying expectations about telehealth limitations versus benefits yielded three telehealth staffing patterns following the trough of disillusionment: complete collapse, recovery to stable levels, or bypassing the trough entirely. This study provides a customisable framework, offering evidence-based guidance for successful telehealth implementation.

## Introduction

**Fig. 1:**
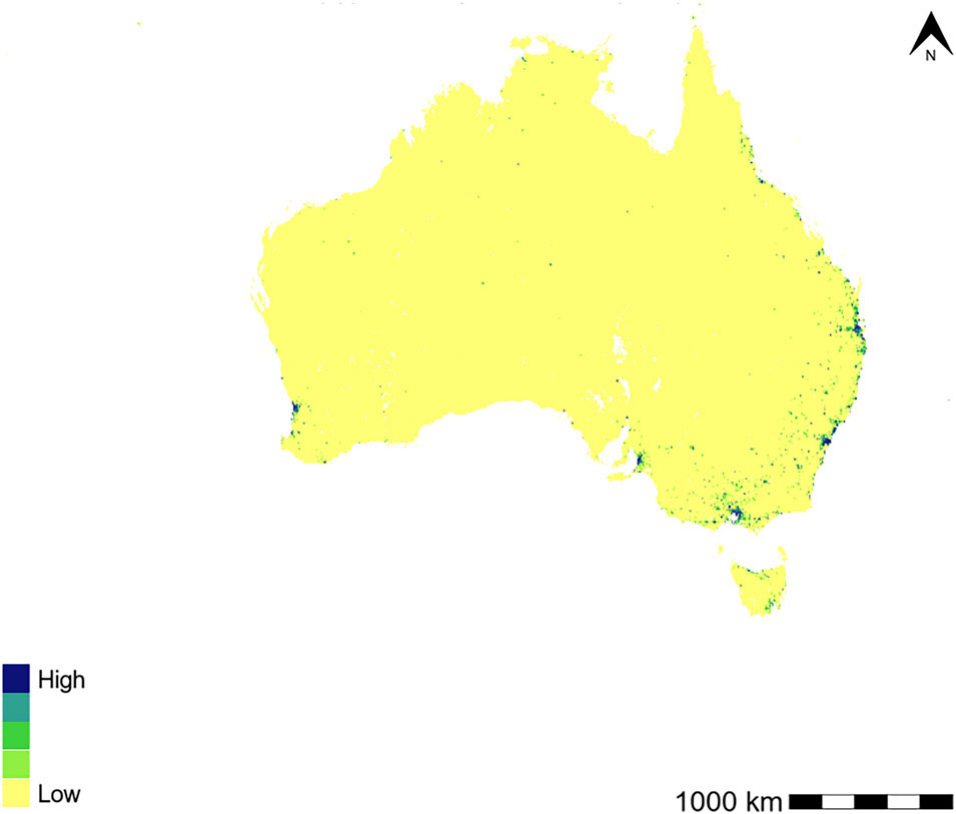
Australia is sparsely populated with the majority of the population living in metropolitan areas along the coastline. The map shows the spatial distribution of population density in 2020 based on country total adjusted to match the corresponding UNDP estimate^45^.

Persistent workforce shortages in rural Australia have created significant challenges in healthcare access in these areas, particularly specialised and critical care services^1–3^. As a result, rural Australians travel or may need to be transported long distances to access specialised healthcare^4^ - See Fig. 1.

Clinician-to-clinician emergency telehealth services, typically operating from rural emergency care facilities where remote specialists support local on-site practitioners, have been widely implemented to address these service gaps. While these services provide rural clinicians with increased access to specialist expertise and reduce professional isolation^5^, they also introduce challenges such as increased workload due to multitasking requirements^6^, increased medicolegal burden^7–10^, and reduced opportunities for tacit knowledge sharing between clinicians^11^, potentially impacting the long-term sustainability of the rural medical workforce. Although these challenges have significant implications for workforce sustainability, previous research on rural telehealth implementation has primarily focused on identifying barriers and facilitators to telehealth adoption^12–14^. Despite a growing body of literature, current research has not analysed how telehealth implementation decisions evolve over time and what impact telehealth has on rural workforce recruitment and retention. The dynamic interplay between evolving perceptions of telehealth, changing resource allocation decisions, and their long-term effects on workforce sustainability remains understudied.

**Fig. 2:**
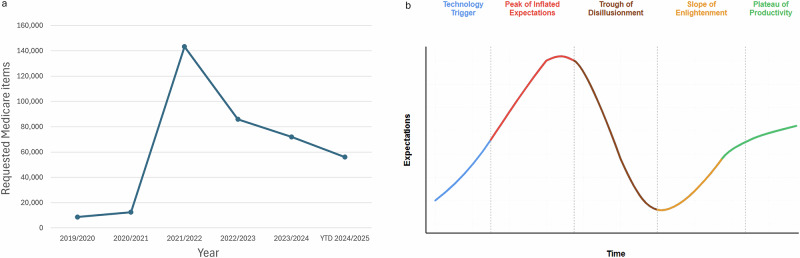
Telehealth adoption patterns in Australia follow the Gartner Hype Cycle. **a** Number of services provided for a single Medicare telehealth item (92024) from 2019/2020 to YTD 2024/2025 across Australia, demonstrating a trajectory that mirrors the hype cycle model. Following a rapid rise during the Technology Trigger phase, utilisation reached a Peak of Inflated Expectations in 2021/2022, then declined to current levels consistent with the Trough of Disillusionment phase. Data obtained from Services Australia^46^. **b** The Gartner Hype Cycle consists of five phases: Technology Trigger, Peak of Inflated Expectations, Trough of Disillusionment, Slope of Enlightenment, and Plateau of Productivity. Adapted from ref. ^16^.

Telehealth has long served rural Australian communities, however, the COVID-19 pandemic catalysed unprecedented adoption across the country, a trend that has since slowed^15^. Although the rate of adoption has slowed, this pattern mirrors the Gartner Hype Cycle (GHC) (See Fig. 2a), which describes how emerging technologies typically progress through phases of enthusiasm, disillusionment, and stabilisation^16^. The cycle begins with a *Technology Trigger* and rapid uptake culminating at the *Peak of Inflated Expectations*, followed by a *Trough of Disillusionment* as limitations emerge, before potentially climbing a *Slope of Enlightenment* towards a *Plateau of Productivity* where realistic benefits and limitations are understood (Fig. 2b).

While the GHC has proven useful in studying technology adoption cycles^17–19^, including telehealth^20^, it has primarily been used as a descriptive tool rather than an operational model that elucidates the underlying mechanisms generating these characteristic patterns. Thus, the GHC falls short in explaining why some technology evolution cycles recover from the disillusionment phase while others collapse^17^. Moreover, it has not been integrated with workforce dynamics, nor applied to examine how evolving perceptions of telehealth may affect rural healthcare staffing patterns over time.

System dynamics (SD) methodology offers an ideal approach for transforming the GHC into an adaptive and adjustable model of rural telehealth implementation, as it enables the study of complex systems behaviour over time by analysing causal relationships, feedback mechanisms, time delays, and accumulations^21,22^. Particularly, it helps to model how perceptions of telehealth implementers evolve and influence resource allocation decisions, and how these decisions subsequently may affect staffing patterns and workforce sustainability. By modelling the relationships between telehealth implementers’ decisions and workforce sustainability in rural areas, along with lags in their perception of telehealth benefits and limitations, SD enables the generation of the characteristic hype cycle pattern from within the system itself.

This study contributes to the body of knowledge of both telehealth implementation and technology adoption through two primary objectives. *First*, building an SD model that operationalises the GHC to understand how clinician-to-clinician telehealth implementation in rural emergency care facilities impacts on-site recruitment and retention. By modelling how telehealth utilisation affects the medicolegal burden of on-site staff, tacit knowledge transfer opportunities, and clinical support access, we aim to identify conditions under which telehealth might enhance rural on-site workforce sustainability. *Second*, extending the GHC from a descriptive tool to a dynamic model by capturing the underlying mechanisms generating its characteristic pattern. By modelling how perceptions of telehealth benefits and limitations evolve and influence resource allocation decisions, we aim to explore potential variations in implementation trajectories and discover critical thresholds determining whether telehealth implementation recovers from the disillusionment phase. Below, we present the results of equilibrium testing, reference mode testing, univariate testing, and multivariate sensitivity analysis. Detailed descriptions of the methods we followed to conduct these tests/analyses appear at the end of this article (See the Methods section).

## Results

### Equilibrium testing

When initialised with inflows equalling outflows, the model maintained equilibrium throughout the 120-month simulation period, confirming the structural soundness and internal consistency of the model.

### Reference mode testing

**Fig. 3:**
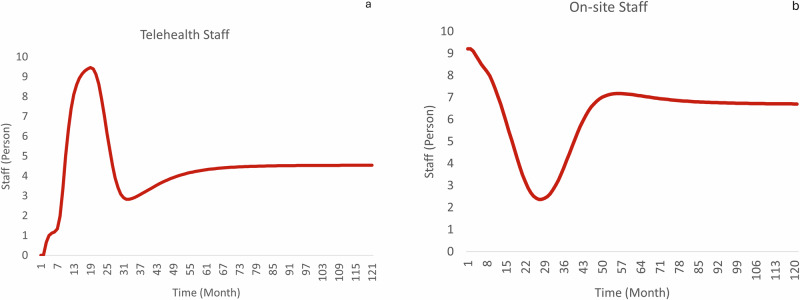
Reference mode testing results. The model successfully produced the expected behaviour over time for both telehealth staffing and on-site staffing. **a** Telehealth staffing behaviour over time follows Gartner’s Hype Cycle where numbers initially rapidly increase to a peak value, followed by rapid collapse before stabilising at a sustained value at the plateau of productivity. **b** On-site staffing behaviour over time shows the inverse pattern of Gartner’s Hype Cycle with an initial rapid decline, followed by recovery before stabilising at a sustained level. On-site staffing declined more gradually than telehealth staffing increased due to differences in the hiring and departure timeframes between telehealth staffing and on-site staffing.

When we introduced 10% of telehealth capacity (Initial On-site Capacity Fraction (IOCF) = 0.9), the model successfully produced all five phases of the GHC in telehealth staffing levels (Fig. 3a): an initial rapid increase (Technology Trigger), followed by a peak (Peak of Inflated Expectations), then a significant decline (Trough of Disillusionment), gradual recovery (Slope of Enlightenment), and eventual stabilisation (Plateau of Productivity). This characteristic pattern emerges from the underlying feedback structure of the system as discussed below.

The initial rapid increase during the technology trigger phase was primarily caused by perceived telehealth benefits driving aggressive resource allocation toward telehealth positions in early stages, reflecting the model’s temporal asymmetry (differences in timing between when benefits and limitations are perceived/recognised), with telehealth benefits being perceived more quickly than its limitations. These perception dynamics created two competing feedback processes. Initially, a reinforcing loop (a feedback process that amplified telehealth utilisation) emerged where perceived benefits of telehealth shifted capacity allocation toward telehealth positions, which further enhanced perceived benefits, driving further utilisation. Subsequently, a balancing loop (a feedback process that counteracted and stabilised this growth in telehealth utilisation) constrained further expansion: recognised limitations restricted additional capacity allocation towards telehealth, stabilising telehealth utilisation.

This dual dynamic simultaneously reduced on-site staffing capacity allocation by shifting capacity away from on-site roles toward telehealth positions via the telehealth benefits driven loop. When the limitation loop subsequently constrained telehealth utilisation, on-site staffing recovered, creating an inverse pattern in on-site staffing levels (Fig. 3b). During these early phases, on-site staffing declined more gradually than telehealth staffing increased due to differences in the hiring and departure timeframes between telehealth staffing and on-site staffing; telehealth positions responded quickly due to shorter hiring timeframes, while on-site positions changed gradually through considerably longer tenures of on-site staff.

A critical dynamic emerged during the disillusionment phase. As enthusiasm decayed and perceived limitations accumulated, the recognition of telehealth limitations triggered a reversal in capacity allocation, with the system attempting to restore on-site capacity while reducing telehealth utilisation. However, the different time delays in telehealth versus on-site staffing processes created asymmetric recovery patterns, as observed in the technology trigger phase. These differences generated patterns where telehealth staffing declined sharply before gradual recovery and plateau, compared to on-site staffing which showed considerably slower recovery toward sustainable levels. While telehealth staffing reached a plateau, it never recovered to its peak value, reflecting an overcorrection by the system in fixing perception issues. Notably, total capacity across both staffing modes also never reached target total capacity (20 in our hypothetical case) due to the differences in delays (hiring, joining, and departing) between on-site and telehealth staffing processes. When we accounted for projected staff losses due to these delays in recruitment planning horizons, the system achieved capacity closer to target capacity, suggesting that understanding system delays is crucial for effective staffing planning and management.

### Sensitivity analysis

In sensitivity analysis (refer to the Methods section below), Limitations Relative to Benefits (LRB), Initial On-site Capacity Fraction (*IOCF)*, and Target Total Capacity (*TTC)* emerged as the parameters exerting the most influence on both on-site and telehealth staffing. Each parameter exerted its influence through multiple feedback mechanisms as discussed below.

#### Limitations relative to benefits (LRB)

The LRB proved most influential as it altered the balance between competing feedback processes that either amplified or counteracted increased telehealth utilisation in the system. When changing its value both +10% and −10% from its baseline value (0.7), asymmetric responses emerged. When LRB was decreased, making limitations less influential relative to benefits, it weakened the balancing loop that counteracts increased telehealth utilisation by pulling capacity allocation back toward on-site staffing after initial telehealth enthusiasm. This allowed the benefits-driven reinforcing process, which amplifies telehealth utilisation, to sustain telehealth levels beyond typical hype cycle patterns. Most critically, the higher telehealth levels activated the attractiveness of rural on-site positions sub-model (See the Methods section below), where two reinforcing mechanisms reduced the attractiveness of on-site positions. These mechanisms operated via reduced knowledge transfer and increased medicolegal burden as consequences of increased telehealth use, leading to lower on-site staffing levels and thereby creating further reliance on telehealth in a reinforcing vicious cycle.

Conversely, when LRB was increased, making limitations more influential, the primary effect strengthened the balancing loop that counters increased telehealth utilisation through limitations recognition, creating sharper reversals in telehealth staffing. However, this balancing effect operated primarily through the limitation perception balancing loop, with reduced impact on the attractiveness of rural positions sub-model and the reinforcing mechanisms that further affected on-site staffing. The asymmetric response pattern, where decreasing LRB had stronger effects (i.e., Mean Absolute Deviation (MAD) 3.11) on on-site staffing levels than increasing it (MAD 0.99), demonstrates how the model structure creates easier pathways toward telehealth dependence once the attractiveness of rural positions sub-model is activated via increased telehealth use.

#### Initial on-site capacity fraction (IOCF)

The sensitivity of the system to *IOCF* reflects its role as the primary trigger pushing the system out of equilibrium, with asymmetric responses emerging from the interaction between benefit perception and capacity allocation feedback mechanisms due to path-dependent positioning within the technology hype cycle.

When IOCF was increased by 10% from its baseline value of 0.9–0.99 (99% on-site, 1% telehealth), the system started at the technology trigger phase with virtually no telehealth experience. This minimal baseline of telehealth capacity created optimal conditions for dramatic benefit perception growth, as every telehealth use generated steeper perception gains at these low usage levels, whilst the hype factor operated with maximum impact. With no limitations yet encountered and substantial room for benefit perception growth, the reinforcing feedback loop that reallocates capacity based on perceived telehealth benefits operated with full intensity, allowing unrealistic expectations to drive dramatic increases in telehealth utilisation.

Conversely, when IOCF was decreased by 10% to 0.81 (81% on-site, 19% telehealth), the system began with a considerable telehealth allocation, leaving limited room for dramatic benefit perception increases. Furthermore, the system started closer to encountering constraints such as increased medicolegal burden and reduced knowledge transfer opportunities as consequences of increased telehealth utilisation, with these balancing feedback mechanisms positioned to constrain dramatic changes in telehealth utilisation. This asymmetric behaviour demonstrates how initial conditions alter the system’s trajectory through the hype cycle, with small parameter changes creating qualitatively different adoption patterns.

#### Target total capacity (TTC)

While no asymmetry in response was observed when changing its value by ±10% from its baseline value (20), TTC emerged as the third most influential factor on both on-site and telehealth staffing levels by scaling the magnitude of all workforce dynamics in the system. This influence can be attributed to the following underlying dynamics. First, larger facilities (higher TTC values) generate proportionally more telehealth usage through expanded telehealth staff, driving stronger benefit perception effects that create more capacity allocation shifts toward telehealth, further driving telehealth utilisation. Second, workforce sustainability mechanisms also operate with greater intensity in larger facilities, where position attractiveness changes affect larger staff pools, creating greater recruitment and retention impacts that feed back into staffing levels. Conversely, smaller facilities experience more constrained dynamics where the same feedback mechanisms operate within tighter bounds, limiting the magnitude of system response.

### Univariate sensitivity testing

Our univariate analysis of *LRB, IOCF*, and *TTC* revealed critical thresholds that function as tipping points in the system, where small parameter changes trigger fundamentally different long-term behaviour patterns. These thresholds help explain the asymmetric sensitivity patterns identified earlier.

#### Limitations relative to benefits (LRB)

**Fig. 4:**
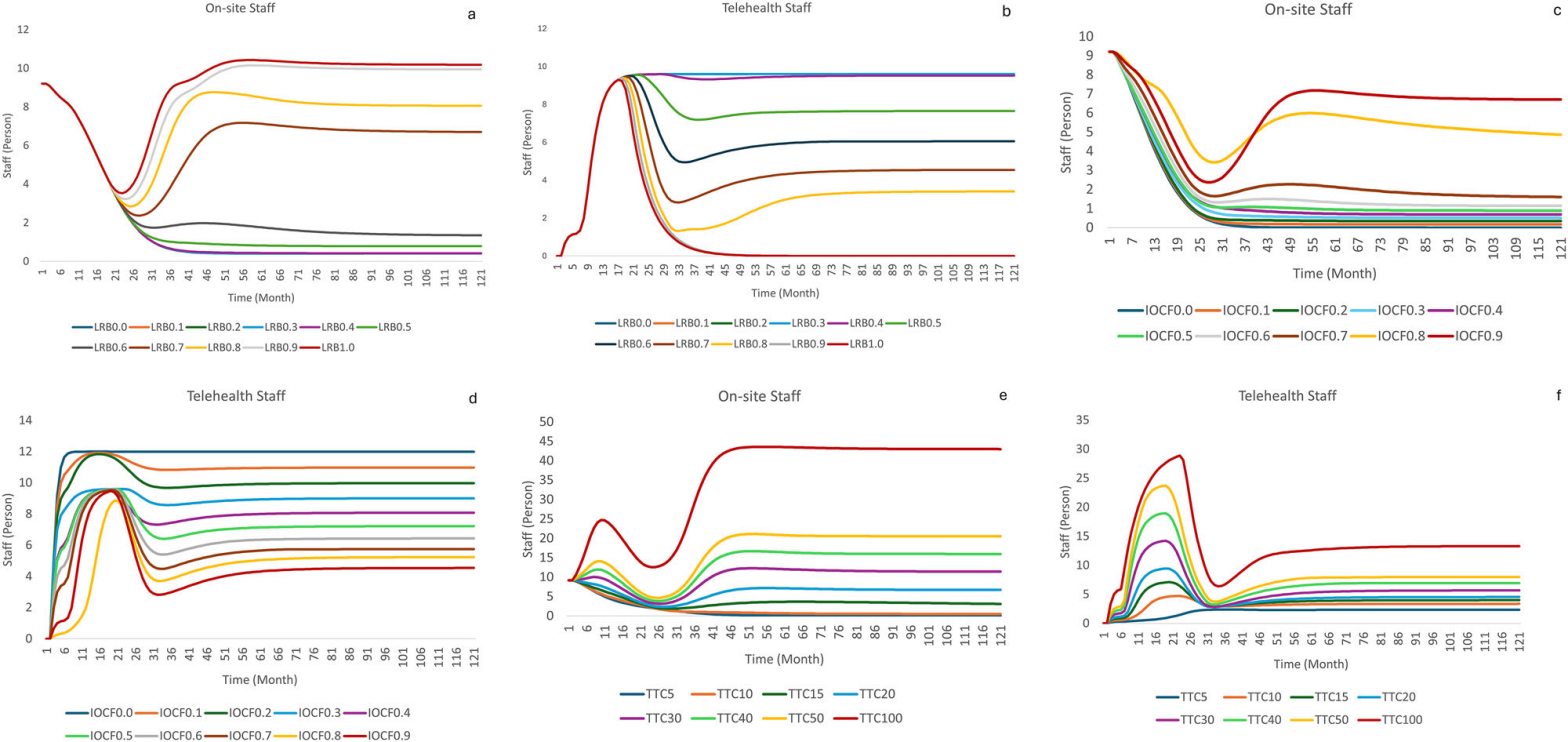
Univariate sensitivity testing results. **a** Behaviour over time for on-site staffing when changing Limitations Relative to Benefits (LRB) across its full range. **b** Behaviour over time for on-site staffing when changing Limitations Relative to Benefits (LRB) across its full range. **c** Behaviour over time for on-site staffing when changing Initial On-site Capacity Fraction (IOCF) across values 0.0–0.9. **d** Behaviour over time for on-site staffing when changing Initial On-site Capacity Fraction (IOCF) across values 0.0–0.9. **e** Behaviour over time for on-site staffing when changing Limitations Relative to Benefits (TTC) across a range of values (5, 10, 15, 20, 30, 40, 50, and 100). **f** Behaviour over time for on-site staffing when changing Limitations Relative to Benefits (TTC) across a range of values (5, 10, 15, 20, 30, 40, 50, and 100).

We identified critical thresholds in LRB value, determining whether reinforcing or balancing loops dominate the system behaviour, and thereby on-site and telehealth staffing levels. This threshold created distinct behavioural patterns that differed between staffing types. For on-site staffing (Fig. 4a), two clear patterns emerged: a) LRB ≤ 0.6 resulted in collapsed on-site staffing that remained in the disillusionment trough with minimal recovery, and b) LRB > 0.6 enabled on-site staffing levels to recover following the disillusionment phase through successful limitation perception feedback that reversed telehealth growth and restored on-site capacity.

For telehealth staffing (Fig. 4b), three distinct patterns were observed: a) very high LRB (0.9–1.0) created such strong limitation perception feedback that telehealth experienced rapid decline into deep disillusionment after the initial peak of inflated expectations; b) low LRB (0.0–0.4) weakened limitation perception processes such that telehealth staffing bypassed the disillusionment phase entirely, remaining at the peak of inflated expectations with sustained high utilisation; and c) moderate LRB (0.5–0.8) allowed the full hype cycle pattern with both benefits and limitation perception feedback mechanisms operating, resulting in telehealth progressing through all phases from trigger through peak expectations, disillusionment, and recovery to balanced productivity plateau.

#### Initial on-site capacity fraction (IOCF)

When we changed *IOCF* across its range of operational values (0.0–0.9), two clear patterns emerged for on-site staffing (Fig. 4c): a) IOCF ≤ 0.7 resulted in collapsed on-site staffing that remained in the disillusionment trough with minimal recovery, experiencing continuous decline toward zero as workforce detrimental feedback structures of reduced knowledge transfer and increased medicolegal burden operated with sufficient intensity to prevent recovery, and b) IOCF > 0.7 enabled on-site staffing levels to recover following the disillusionment phase, as these detrimental feedback mechanisms were not yet operating with sufficient intensity to prevent on-site staffing recovery to sustained productive levels.

For telehealth staffing (Fig. 4d), the analysis revealed patterns influenced by the minimum on-site capacity constraint of 0.2 described in the methods. IOCF values above this constraint all converged to the same peak level during the inflated expectations phase as benefit perception feedback loops drove capacity allocation with maximum impact, demonstrating how the constraint capped maximum telehealth capacity. These constrained scenarios then followed similar hype cycle trajectories with disillusionment troughs of varying degrees before recovering to different plateau levels as balancing feedback mechanisms from telehealth limitations restored system balance. When the constraint was removed for testing IOCF 0.1, it showed an unconstrained peak as reinforcing benefit perception loops operated without capacity limits before experiencing disillusionment and plateau, while IOCF 0.0 represented the scenario with all telehealth capacity with no on-site staffing.

#### Target total capacity (TTC)

Univariate testing of TTC confirmed our sensitivity analysis findings that identified TTC as a key scaling factor for all workforce dynamics in the system. Starting from an initial capacity below 10 with IOCF maintained at 0.9 across all scenarios, the patterns reveal how facility size may shape telehealth implementation dynamics.

For on-site staffing (Fig. 4e), higher TTC values (30, 40, 50, 100) demonstrated initial expansion phases as the system scaled up to meet new capacity targets, followed by substantial declines as capacity allocation shifted toward telehealth driven by perceived benefits, deeper troughs as rural position attractiveness deteriorated through reduced knowledge transfer opportunities and increased medicolegal burden on remaining on-site staff (effects that were more pronounced in larger facilities with greater staff pools), and eventual recovery as telehealth limitations became apparent and the balancing feedback loop constrained telehealth utilisation and restored on-site staff capacity. Lower TTC values (10, 15, 20) showed more constrained dynamics with modest initial changes and gradual declines. Even though these scenarios started below target capacity, on-site staffing never peaked because perceived benefits of telehealth reinforcing loops drove telehealth utilisation and brought on-site staffing to levels lower than initial on-site capacity, with TTC values of 15 and below never recovering. The smallest capacity values (TTC = 5, 10) proved particularly vulnerable, experiencing continued decline without recovery, indicating system instability at very small scales.

For telehealth staffing (Fig. 4f), higher TTC values (30, 40, 50, 100) enabled pronounced hype cycle behaviours with dramatic peaks during the inflated expectations phase, substantial troughs during disillusionment, and recovery to higher plateau levels. Lower TTC values (10, 15, 20) constrained these dynamics to modest fluctuations with lower peaks and plateau levels. Notably, TTC = 5 never followed the hype cycle remaining at minimal levels throughout the simulation.

### Multivariate sensitivity testing

**Fig. 5:**
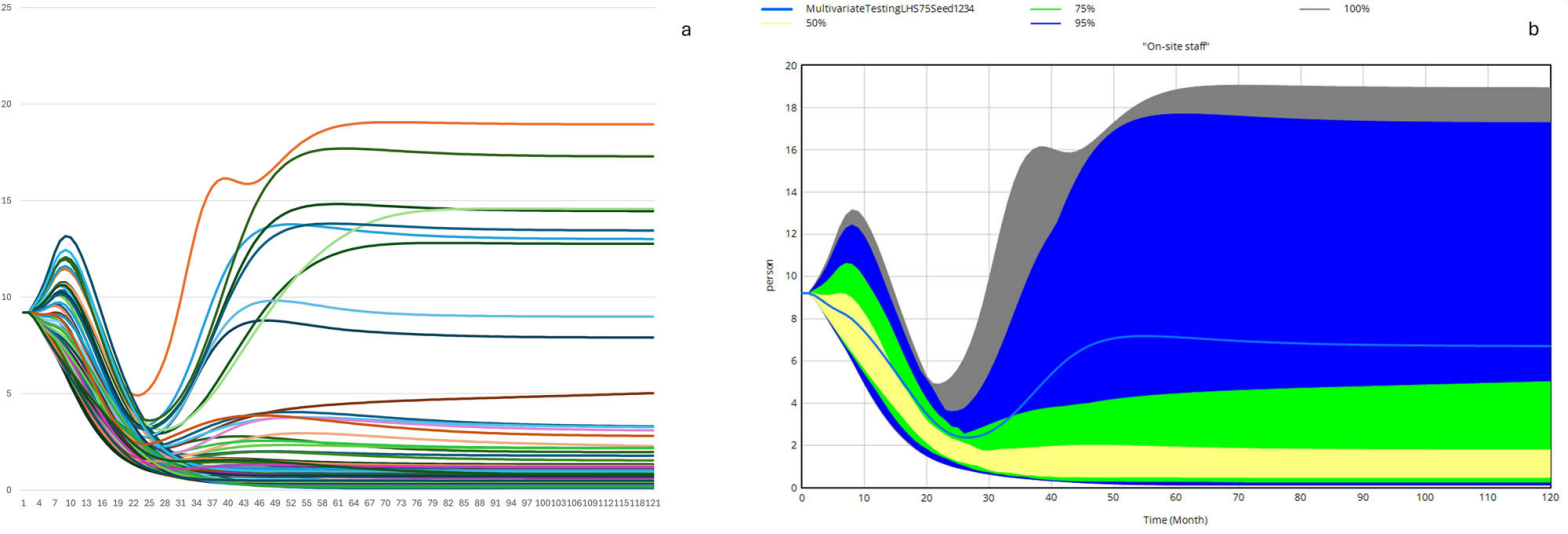
Multivariate sensitivity testing results. **a** Individual trajectories of on-site staffing for all 75 simulation runs. **b** Confidence bounds of on-site staffing for 75 simulation runs.

Analysis of on-site staffing levels across multiple simulation runs confirmed the patterns identified in univariate testing and sensitivity analysis. All runs showed similar declining on-site staffing trajectories during the initial phase (Fig. 5a), though these varied with some initially increasing potentially indicating higher target total capacity than initial starting on-site capacity as the system worked across both modalities to reach target levels before perceived telehealth benefits reinforcing mechanisms brought staffing to lower levels. However, a critical bifurcation emerged during the middle phase where the confidence bands began to separate (Fig. 5b), reflecting the activation of competing feedback mechanisms we identified earlier as limitation balancing loops and rural position attractiveness dynamics began countering benefit-driven capacity shifts.

This divergence is most evident in the wider 95% and 100% confidence bounds (blue and grey regions in Fig. 5b), which show pronounced separation between upper and lower trajectories when balancing mechanisms attempt to counter telehealth expansion with varied degrees of intensity depending on starting conditions as well as the degree to which the reinforcing mechanisms of reduced knowledge transfer and increased medicolegal burden are operating in the attractiveness of rural positions sub-model, thus influencing whether on-site staffing levels recover during the trough of disillusionment phase following decline during the technology trigger or they continue in decline. While the 50% confidence bound shows less dramatic separation, the overall pattern across all confidence bounds confirms the existence of dual steady states in on-site staffing, demonstrating how the system’s embedded thresholds create fundamentally different long-term trajectories depending on which reinforcing loop ultimately dominates.

To understand the distribution of system behaviours within the parameter space, we classified these runs into two categories based on their behaviour following the trough of disillusionment (Refer to Supplementary Data S1). Recovered runs were those showing strong recovery and maintained on-site staffing levels of at least 20% of the target total capacity, indicating that balancing mechanisms successfully countered the benefits-driven reinforcing loop. Failed runs were those showing minimal on-site staffing levels less than 20% of total target capacity or near zero, reflecting scenarios where workforce sustainability feedback loops created cascading effects that reinforced telehealth dependence. This classification aligns with the constraint we implemented in the model that the initial on-site capacity should not be less than 20%, ensuring our definition of failure or recovery aligns with initial implementation plans. It also differentiates between small facilities where target capacity is small (thus the system will show moderate recovery) compared to large capacity facilities where the system works aggressively to reach larger capacity, resulting in strong recovery under ideal parameter conditions.

**Fig. 6:**
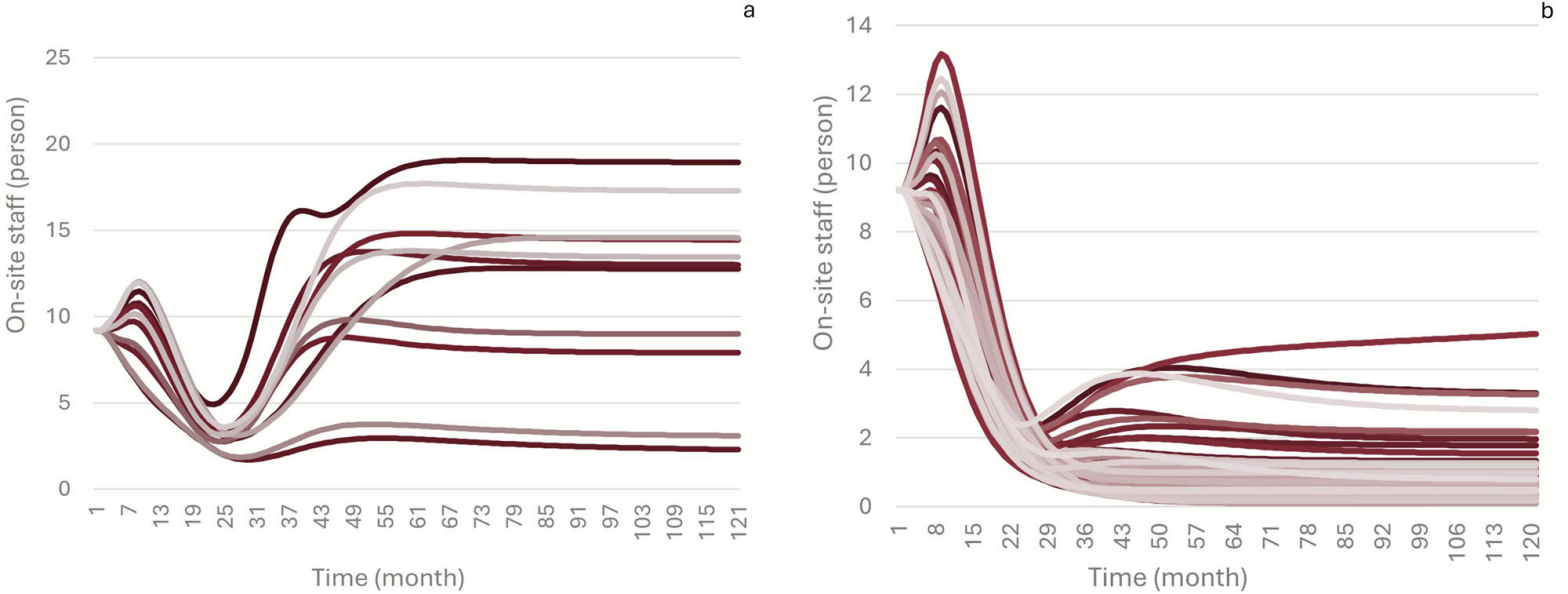
Trajectories of simulated scenarios classified as recovered or failed. **a** Scenarios where on-site staffing recovered following the disillusionment phase (15%, 11/75). **b** Scenarios where on-site staffing failed to recover following the disillusionment phase (85%, 64/75).

Remarkably, of the 75 simulation runs, only 11 (15%) recovered following the disillusionment phase (Fig. 6a), while 64 (85%) failed to recover (Fig. 6b). This distribution confirms our earlier finding that the model structure creates easier pathways toward telehealth dependence, with most parameter combinations activating the self-reinforcing cycles that compromise on-site staffing levels through a) direct capacity reallocation to telehealth and b) indirect erosion as increased telehealth utilisation reduces rural position attractiveness through decreased knowledge transfer and increased medicolegal burden. The predominance of failed recovery scenarios (85%) reflects how reinforcing loops driving telehealth expansion typically overwhelm balancing loops attempting to preserve workforce sustainability.

**Fig. 7:**
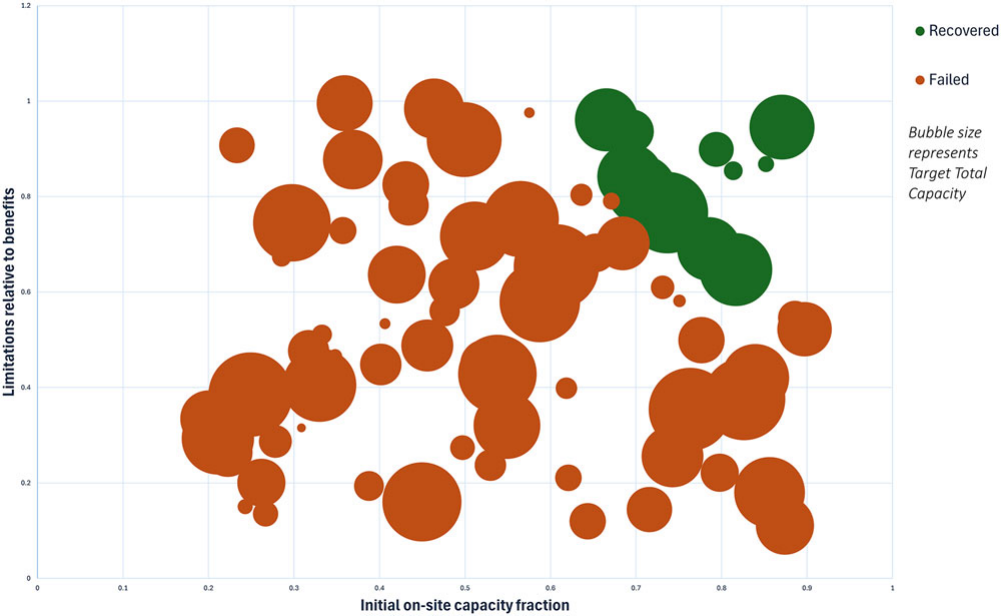
Parameter combinations resulting in recovered versus failed system states.

We further investigated how the three most influential parameters from our sensitivity analysis impact on-site staff levels by creating a three-dimensional scatter plot to identify if there exists an ideal parameter space where rural clinician-to-clinician telehealth would be more likely to maintain sustainable on-site staffing. Figure 7 shows all simulated runs with bubble size representing *TTC*, x-axis *IOCF*, and y-axis *LRB* for the corresponding run. Recovered runs clustered predominantly in the upper right quadrant of the parameter space (Green bubbles), characterised by high IOCF values (≥0.7) combined with high *LRB* values (≥0.6). This clustering directly validates our threshold analysis, confirming that recovery requires both starting above the critical *IOCF* threshold where benefits-perception dynamics operate with less intensity and maintaining sufficient *LRB* values to activate limitation-perception balancing mechanisms during the disillusionment phase.

## Discussion

Our model makes several theoretical contributions by conceptualising the GHC as an endogenous structure in the rural clinician-to-clinician emergency telehealth system. We transformed the GHC into a dynamic operational model where the characteristic pattern of enthusiasm, disillusionment, and eventual stabilisation emerges from temporal asymmetries in how benefits and limitations are recognised. By doing so, our work extends the traditional GHC to identify multiple potential trajectories in telehealth patterns rather than a single universal pattern, in alignment with previous research that criticised the GHC for reflecting only one possible scenario of technology adoption^17,23^.

Our findings challenge the implicit assumption in the traditional GHC that all technology implementations experience a trough of disillusionment followed by recovery. We observed three distinct trajectories determined by the parameter *LRB*. *The Collapse Trajectory* (0.8–1) where systems experience such severe disillusionment that telehealth adoption completely collapses, never recovering from the trough, representing a failure pattern not captured in the traditional Hype Cycle model. *The Recovery Trajectory* (0.5–0.8) follows the classic hype cycle pattern, experiencing a distinct trough of disillusionment followed by recovery to intermediate levels. *The Bypass Trajectory* (0.0–0.4) where systems essentially bypass the trough of disillusionment entirely, maintaining peak telehealth adoption levels without significant correction, representing an overly optimistic implementation pattern.

Our model of rural telehealth captures several key features of complex adaptive systems that enhance our understanding of telehealth implementation dynamics. For instance, the identification of bifurcation points and alternative stable states extends complex systems theory in healthcare^24–26^ by demonstrating how differences in initial conditions can lead to qualitatively different trajectories creating path dependencies that constrain future options^27,28^. They also exemplify the system archetype “success to the successful”^29^, where early telehealth implementation decisions may create irreversible trajectories in both telehealth and on-site staffing levels.

Our findings have implications for telehealth practice and policy. The strong clustering of recovered runs at high IOCF and LRB suggests that successful rural telehealth implementation requires moderately conservative initial telehealth allocation and realistic expectations about its limitations weighed against benefits. Moreover, the small proportion of scenarios yielding recovery of on-site staffing in our tested parameter space (15%) highlights the dynamical complexity and challenges in sustaining on-site staffing when implementing rural clinician-to-clinician emergency telehealth within the conditions modelled.

While initial telehealth implementation typically emerges from administrative levels, our model demonstrates that long-term sustainability depends on maintaining balanced perceptions influenced by frontline clinicians through increased usage and experience. This is exemplified by the strong influence of *LRB* in determining success in sustaining on-site staffing. Thus, expectation management during initial implementation stages is crucial. Overly optimistic views can undermine implementation, however, if frontline staff perceive telehealth as inherently problematic rather than value-adding, that can also potentially trigger full reversal from telehealth. These align with Wade et al.’s findigns^30^, who concluded that clinician acceptance is crucial for sustaining telehealth, and with Armenakis and Harris^31^, who positioned stakeholders’ beliefs about beneficial change as a critical determinant of implementation success. What’s more, the delayed differentiation between system trajectories when changing *LRB* suggests that early stages may provide false reassurance, emphasising the need for continuous monitoring and adaptive approaches to evaluation^25,32,33^.

Furthermore, sustaining healthcare innovations such as telehealth requires a culture of health equity where ownership of the implemented innovation is embedded across all health system levels, from leadership to frontline clinicians^34^. The three distinct patterns we observed in our model (collapse, recovery, or bypass of the trough of disillusionment) likely correspond to varying degrees of systematic ownership, as moderated through *LRB*. These insights raise fundamental questions about long-term telehealth sustainability in rural areas in terms of integration of recruitment processes to account for both the benefits and limitations of telehealth and the redefinition of telehealth’s role from merely a service delivery modality to a rural workforce capacity-building model, with mechanisms for reinvesting realised savings from telehealth utilisation into rural workforce development. This would create an ecosystem where telehealth not only addresses immediate workforce gaps but actively strengthens rural workforce capacity long-term. Without addressing these systematic issues, even technically sound telehealth implementation remains vulnerable to the sustainability challenges our model demonstrates. Policymaking efforts should therefore prioritise embedding rural workforce sustainability as a core organisational responsibility throughout telehealth implementation and governance processes.

Our analysis revealed that facility size acts as a critical moderating variable, with larger facilities demonstrating greater resilience when other parameters approach critical thresholds. Smaller facilities recover less effectively from suboptimal implementation conditions, likely because such facilities typically operate with minimal redundancy^35^, where losing even one on-site practitioner creates disproportionate impacts knowledge sharing and workload. Conversely, medium and large facilities demonstrate greater capacity to absorb the temporary disruptions associated with the disillusionment phase due to having enough on-site staff to maintain operational continuity and knowledge transfer opportunities. Implementation policies should acknowledge these fundamentally different dynamics across facility sizes rather than applying a one-size-fits-all approach.

A key workforce planning insight revealed that reactive hiring consistently yields below-target staffing levels. To rectify this gap between target and actual capacity, delays in both the hiring and joining processes should be accounted for when planning recruitment; necessitating workforce planning that anticipates departures based on historical patterns rather than responding to vacancies. These implications are summarised with implementation guidelines and recommendations in Table 1.

**Table 1:** Policy and practice recommendations for clinician-to-clinician telehealth implementation in rural healthcare settings

Issue	Recommendation	Implementation guidelines
Initial implementation decisions	Start with a moderately conservative initial allocation of telehealth to provide greater resilience during the disillusionment phase.	• Initial on-site capacity at ≥70% (limit initial telehealth to ≤30% of total capacity) • Implement in phases with clear evaluation criteria • Start with specific clinical areas with strongest evidence • Expand gradually based on outcomes assessment
Perception management	Adopt a realistic yet balanced approach that acknowledges potential limitations from the start while maintaining a positive outlook about telehealth’s value to create a sustainable implementation trajectory.	• Avoid over-selling benefits during initial phases • Provide balanced messaging that acknowledges both potential benefits and limitations • Regular stakeholder feedback to assess evolution of perceptions as implementation progresses.
Facility size considerations	Apply scale-specific implementation strategies with particular attention to smaller facilities, as they may experience disproportionate vulnerability in sustaining on-site staffing following telehealth implementation	• Start with more conservative telehealth allocation in smaller facilities • Provide additional support resources for smaller facilities • Offer targeted special workforce incentives for smaller sites
Workforce planning	Implement proactive recruitment with calibrated planning horizons accounting for delays in the hiring and onboarding processes to achieve capacity targets.	• Anticipate departures based on historical data • Maintain recruitment pipelines even when fully staffed • Account for hiring and joining delays in capacity planning
Implementation monitoring	Apply continuous monitoring of early warning indicators during critical transitions after the disillusionment phase.	• Track staffing levels and position attractiveness as lead indicators • Establish early monitoring mechanisms to track telehealth’s impacts on tacit knowledge transfer, medicolegal burden, and clinical support. • Define specific thresholds for intervention • Implement regular review cycles
Knowledge transfer mechanisms	Establish formal knowledge transfer systems to compensate for reduced incidental learning due to implementing telehealth	• Establish structured mentoring programs • Offer protected time for professional development • Establish virtual learning communities • Maintain regular on-site specialist visits to complement telehealth
Medicolegal burden management	Apply mitigation strategies to reduce medicolegal burden on on-site staff due to implementing telehealth	• Implement standardised documentation protocols for telehealth consultations • Develop clear clinical governance frameworks that delineate responsibility between on-site and telehealth clinicians • Establish clear escalation pathways for complex cases requiring additional medicolegal oversight
Recovery strategies from suboptimal states	Develop specific interventions for systems in telehealth-dominated mode that struggle to recover to sustainable on-site staffing levels.	• Temporary overshooting of on-site staffing targets to push the system out of the suboptimal state • Offer enhanced incentives for rural on-site positions

In addition to the theoretical and practical contributions discussed above, the comprehensive sensitivity analysis we conducted is a core strength of this study revealing asymmetric response patterns beyond simple parameter rankings. To our knowledge, this is the first dynamical model of telehealth implementation and its impact on workforce sustainability in rural healthcare settings. By making the model publicly available^36^, we enable others to build upon and customise the model for specific implementation contexts.

Another strength of our model is that it is agnostic to initial adoption drivers of telehealth, focusing on the subsequent implementation dynamics rather than initial trigger mechanisms, whether being policy mandates (such as was the case with COVID-19 policy mandates) or technology enthusiasm and need. This characteristic makes our model applicable across diverse contexts, as the model examines how organisations navigate expectation management and capacity planning regardless of what initially prompted telehealth adoption. The three trajectory patterns of telehealth captured by our model (i.e., collapse, recovery, bypass) represent dynamics that operate independently of the initial driver of adoption.

Despite discussed strengths and contributions, our model has several limitations. First, the parameter values used in testing are hypothetical rather than empirically derived. Furthermore, our model primarily tracks medical staff dynamics in rural emergency settings, potentially limiting its generalisability to other healthcare contexts or groups such as nursing, allied health, or administrative staff. Our model represents a generic rural facility rather than a specific contextualised setting with varied organisational, cultural, and geographical contexts that may introduce additional dynamics not captured in our generalised framework. Additionally, the model assumes a 1:1 substitution ratio between on-site and telehealth capacity. While this was necessary to serve as a baseline for testing, it may oversimplify real-world staffing patterns where one telehealth practitioner might support multiple facilities or where hybrid roles combine on-site and telehealth responsibilities.

These limitations suggest several future directions. First, empirical validation of the model through data collection in rural facilities implementing telehealth would strengthen its predictive capability. Tracking perception metrics, staffing levels, and workforce sustainability indicators over time would refine our understanding of the critical thresholds that determine the system behaviour. Our analysis identified the *LRB* as a key predictor of sustaining staffing levels, which remains a conceptual rather than directly measurable parameter. Future research should focus on operationalising this parameter through longitudinal perception surveys that track how stakeholders’ views evolve throughout the implementation cycle. Furthermore, testing specific policy approaches would advance our understanding of how to mitigate the negative impacts of telehealth on on-site recruitment and retention while preserving its benefits. Examples of potential interventions that can be simulated and tested in the model include structured mentoring programs and rural on-site positions enhancement strategies or incentives (See Table 1 for more recommendations).

In conclusion, our system dynamics model provides insights into the complex interplay between clinician-to-clinician telehealth implementation and on-site workforce sustainability in rural Australian healthcare settings. By operationalising and extending the GHC, we identified critical thresholds and multiple trajectories for staffing levels when implementing telehealth, contributing to both the theory and practice of sustainable telehealth implementation in rural areas. Our model demonstrates that sustaining on-site staffing following telehealth implementation requires careful attention to initial resource allocation decisions, perception management, and consideration of facility size effects. This work establishes a foundational modelling framework that can be customised for diverse healthcare contexts and provides the theoretical basis for future empirical validation studies, enabling researchers and policymakers to test specific interventions and refine implementation strategies through controlled parameter exploration before real-world deployment.

## Methods

### Model conceptualisation

The model conceptualises rural clinician-to-clinician emergency telehealth implementation as a process where resource allocation decisions made by telehealth implementers, influenced by their perceptions of telehealth benefits and limitations, affect on-site medical staffing in rural emergency care facilities. The model posits that the increased utilisation of telehealth affects rural on-site medical position attractiveness through three key mechanisms: increased medicolegal burden on on-site medical staff^7–10^, reduced tacit knowledge transfer opportunities^11^, and changes in access to clinical support and specialised expertise^5^. These factors subsequently influence both recruitment and retention of medical staff for rural on-site positions. Perceptions of benefits, limitations, and position attractiveness that shape telehealth implementation were modelled to adjust gradually rather than instantaneously, reflecting information delays in the system^21,37^.

The model also tracks medical practitioners staffing in rural emergency care facilities experiencing higher turnover rates and greater recruitment challenges. However, it aligns more closely with the perspectives of telehealth implementers, administrators, and policymakers rather than frontline clinicians. This reflects decision-making dynamics in telehealth adoption, where implementation decisions typically originate at administrative levels. Nevertheless, clinicians’ perspectives were incorporated by capturing how insights from frontline experiences gradually influence the perception of telehealth limitations. This perspective justified our model’s asymmetric perception structure, where benefits are recognised more quickly than limitations, reflecting insights from our stakeholder interviews, which suggested that administrators and policymakers typically held more enthusiastic and optimistic views about telehealth compared to the more sceptical clinicians. For example, clinicians primarily highlighted concerns about telehealth’s inefficiency, limited applicability for certain medical conditions, and possible resource redirection away from rural facilities, while administrators and policymakers emphasised telehealth’s potential for cost-effective care delivery, reduced healthcare expenditures, and improved patient access^38^.

Our scope is limited to areas with limited existing emergency specialist capacity, whether delivered through visiting specialists, locum arrangements, or rural generalists with emergency training, with the assumption that telehealth is implemented to supplement and support such services. This excludes areas where telehealth serves as essential service provision as no local alternatives exist. Furthermore, our scope is limited to emergency telehealth services, where the time-critical nature of care, the shared responsibility between the onsite clinician and the remote clinician, and the real-time interactions create unique feedback loops between telehealth and these services that differ from other specialist services with more predictable consultation models.

The model assumes a direct substitution relationship between on-site and telehealth staff, serving as a simplifying assumption to establish a baseline for analysis. This relationship means that telehealth and on-site staff are treated as interchangeable in terms of capacity allocation, though with different characteristics regarding recruitment, retention, and effects on rural position attractiveness.

### Model development

The model structure was grounded in previous qualitative system dynamics work examining the impact of telehealth in rural Australia (Results from this phase are reported elsewhere). This foundational work included one-on-one semi-structured interviews that took place between November 2023 until April 2024 with rural clinicians (*n* = 6); telehealth administrators, policymakers, and not-for-profit representatives (*n* = 8), and telehealth consumers (*n* = 6). In June 2024, two workshops took place, each with a 90-min duration. A total of 16 participants were involved across both sessions with six participants attended both workshops, while twelve were present at the first and ten at the second. The workshops participants consisted of researchers specialising in rural health, telehealth, and health systems research, along with practitioners with clinical experience in rural settings. Additionally, we also systematically synthesised evidence on the impact of telehealth in terms of both unexpected benefits and drawbacks in rural Australia from the published peer-reviewed literature^5^, which informed our approach, and the key assumptions incorporated when developing this model. Building on this work, we adopted the GHC as the theoretical foundation underpinning the modelling process reported here.

### Model structure

**Fig. 8:**
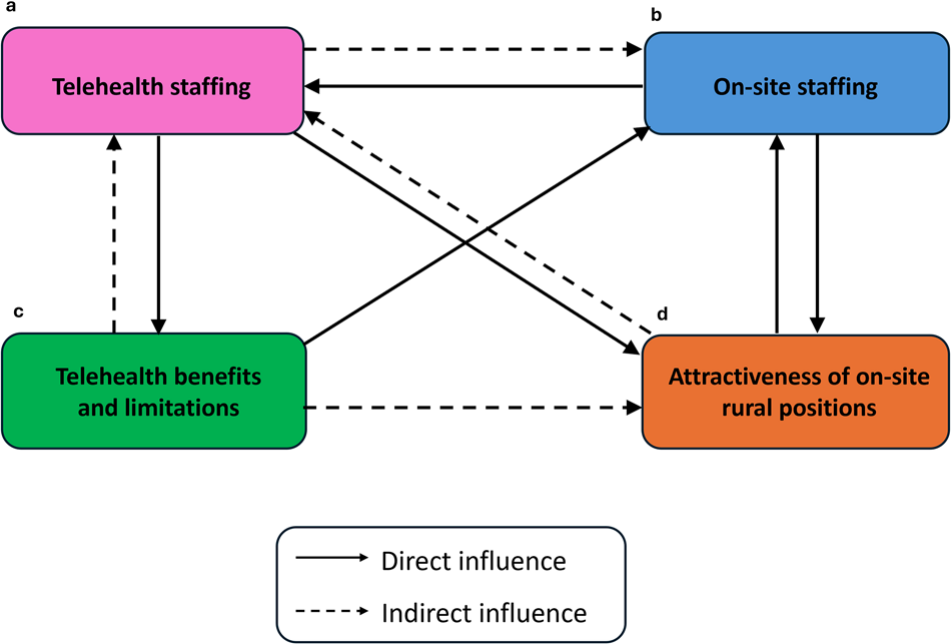
A rural clinician-to-clinician telehealth system dynamics model consisting of four interconnected sub-models. **a** Telehealth staffing, **b** On-site staffing, **c** Telehealth benefits and limitations, and **d** Attractiveness of on-site rural positions. The sub-models interact in several ways both directly and indirectly.

The model was built using Vensim PLE Plus software^39^. The model file and full documentation including variables, descriptions, and equations are provided in supplementary materials. The model consists of four interconnected sub-models, as shown in Fig. 8:

- Telehealth staffing sub-model tracks the flow of telehealth medical staff through recruitment, joining, and departure processes.
- On-site staffing sub-model tracks the flow of on-site medical staff through similar processes.
- Telehealth benefits and limitations sub-model operationalises the Gartner Hype Cycle by capturing how perceptions of telehealth evolve over time.
- Attractiveness of rural on-site positions sub-model models the impact of telehealth on on-site staffing through the three mechanisms identified above.

The sub-models interact in several ways as can be seen from Fig. 8 and detailed below. A direct influence link indicates that variables from one sub-model directly affect or serve as inputs to another sub-model without any intermediary factors. An indirect influence link represents the type of influence that occurs when one sub-model affects another through an intermediate sub-model, rather than having a direct connection.

#### On-site staffing sub-model

The on-site staffing sub-model draws on established workforce planning SD model structures^40,41^. It tracks the levels of two key variables: on-site staff being hired; representing staff in the recruitment pipeline; and active on-site staff. These levels change through three rates: rate of new staff entering the pipeline (on-site staff hiring), rate of hired staff becoming active (on-site staff joining), and rate of active staff leaving (on-site staff departing).

This sub-model incorporates feedback from both the benefits and limitations sub-model and the attractiveness sub-model. The telehealth benefits and limitations sub-model affects allocated on-site capacity, while the attractiveness sub-model influences recruitment timelines (i.e., rate of new staff entering) and staff tenure (i.e., rate of active staff leaving). Lower position attractiveness extends hiring timeframes and shortens the average time staff remain in positions. These feedback mechanisms create self-reinforcing cycles that can either stabilise or destabilise on-site staffing levels.

#### Telehealth staffing sub-model

The telehealth staffing sub-model parallels the on-site structure but reflects telehealth processes, assuming faster recruitment and higher staff turnover. Like its on-site counterpart, it tracks the levels of telehealth staff in the recruitment pipeline and active telehealth staff.

Telehealth staff levels, combined with on-site staff, determine the telehealth staff ratio of total capacity. This ratio drives the three key mechanisms in the attractiveness sub-model: tacit knowledge transfer opportunities, medicolegal implications, and clinical support. This creates a feedback loop where telehealth staffing decisions influence on-site position attractiveness, which affects on-site staffing levels and subsequently influences telehealth staffing.

#### Benefits and limitations sub-model

This sub-model operationalises the GHC by generating its characteristic pattern through three key mechanisms: a) a temporal asymmetry in perception formation where benefits are recognised more quickly than limitations, reflecting the administrative perspective discussed earlier; b) an enthusiasm factor that decays over time means initial perceptions are inflated by enthusiasm that gradually diminishes; and c) an overcorrection factor following limitations recognition, leading to the trough of disillusionment.

The sub-model tracks the levels of perceived telehealth benefits and perceived telehealth limitations as telehealth usage increases, influencing staffing allocation decisions between on-site and telehealth modalities. This creates a feedback loop where initial allocation decisions influence telehealth experiences, which shape perceptions, which in turn affect subsequent staffing decisions.

#### Attractiveness of on-site positions sub-model

This sub-model captures how increased telehealth usage affects the attractiveness of rural medical positions through three mechanisms: a) the medicolegal effect reflects how telehealth increases medicolegal burden on on-site medical staff non-linearly as they carry out tasks on behalf of telehealth specialists; b) the tacit knowledge effect shows how telehealth reduces opportunities for tacit learning, diminishing as on-site staffing reach critical low levels; and c) the clinical support effect, modelled as an inverse U-shape, initially increases with telehealth usage, providing access to specialist expertise, but eventually declines as insufficient on-site staff remain to benefit from this improved clinical support and implement care.

The sub-model tracks the level of perceived attractiveness of rural on-site positions and influences the on-site sub-model through influencing recruitment timeframes and staff tenure, as discussed earlier, creating the primary feedback loop determining on-site staffing levels under different telehealth implementation scenarios.

### A hypothetical case study

The model serves as a customisable framework adaptable to various rural emergency facilities using clinician-to-clinician emergency telehealth services. While the model structure remains broad, parameters can be modified to represent different facility sizes, staffing profiles, and regional characteristics.

Real-world data on telehealth staffing dynamics in rural emergency departments are limited due to the nascent nature of these programs and confidentiality constraints around staffing information. Additionally, the high variability across rural Australian facilities makes it challenging to identify a single representative case. Therefore, we developed a hypothetical case informed by published literature, where possible, on rural healthcare staffing challenges in Australia, workforce shortages, and telehealth implementation.

For demonstration purposes, we developed a hypothetical case based on a medium-sized rural emergency department with a target of 20 medical staff. We assume a minimum on-site medical capacity of 20% is required for viable healthcare delivery and to prevent complete capacity shift towards telehealth as an unintended consequence of rural position attractiveness erosion rather than deliberate policy decisions. The facility faces recruitment challenges for on-site positions due to the limited availability of candidates and relocation requirements for those joining, with 9-month recruitment timeframes, 3-month joining delays, and 18-month average tenure. Telehealth positions have shorter timeframes (3-month recruitment, 1-month joining) and higher turnover (6-month average tenure), reflecting the ease of both entry and exit to these positions due to the absence of relocation barriers.

The timeframes above were selected to represent difference in barriers to entry and exit between telehealth and on-site positions. Acknowledging the limited availability of published literature comparing these timeframes in rural Australian contexts, we conducted sensitivity analysis by varying these timeframes in our model ±25% around the baseline assumptions above. The results of this sensitivity testing show that varying these timeframes had minimal effect on the behaviour of the system, suggesting that the system behaviour emerges from the underlying feedback structure and relationships captured by our model rather than specific timeframe values, and as such we carried out our simulation with the base run values.

This hypothetical case serves as a controlled baseline that enables systematic exploration of the parameter space across diverse rural healthcare contexts. Rather than constraining our analysis to a single real-world facility with fixed characteristics, this approach allows us to examine how varying combinations of key parameters (facility size, initial capacity allocation, and perception dynamics) affect system behaviour across the full spectrum of rural Australian healthcare settings. By establishing this controlled starting point with moderate parameters, we can systematically test how the model responds to more extreme conditions that reflect both the harsher realities faced by many rural facilities and the more favourable circumstances of well-resourced centres. This methodological choice facilitates comprehensive sensitivity analysis and enables identification of critical thresholds that determine system trajectories, as demonstrated in our multivariate testing of 75 parameter combinations below. The hypothetical case thus functions as a customisable framework that can be adapted to represent specific facility contexts while maintaining the analytical rigor necessary to understand the underlying system dynamics. Complete parameter values for the baseline case are provided in Supplementary Materials.

### Model testing

Our testing approach encompassed three key components. First, we conducted equilibrium testing to establish a baseline by verifying the stability of the model when undisturbed, where we initialised various stocks so that inflows equalled outflows and rates of change remained constant. The initialisation values used to achieve equilibrium conditions are detailed in Supplementary Materials. Second, we performed reference mode testing to confirm the model’s ability to reproduce the GHC pattern in telehealth implementation, where reference modes are the specific behaviour patterns that the model aims to explain. Finally, we conducted sensitivity testing to identify which parameters most significantly influenced model behaviour, where we further examined their influence via univariate testing and multivariate testing.

To assess the model’s ability to reproduce the GHC pattern, we disturbed the system from equilibrium by introducing a 10% telehealth allocation with all other parameters kept at their values from equilibrium. This run constituted our base run for all subsequent sensitivity testing. To conduct sensitivity analysis of the three most influential parameters, we varied each parameter by ±10% from its base value and measured its impact on both on-site staffing and telehealth staffing using mean absolute deviation (MAD). We ran these tests in Vensim PLE Plus using the Sensitivty2All function (The results of this test can be found in Supplementary Materials).

To identify the three most influential parameters affecting both on-site and telehealth staffing, we first calculated the average MAD values (MAD values corresponding to a ±10% change in parameter value) for the five most influential parameters for each staffing type. We limited our analysis to five parameters as the remaining parameters showed negligible impact with very low MAD values (MAD < 0.2) across both telehealth and on-site staffing. We then averaged these MAD values across both on-site and telehealth staff and ranked the parameters according to their combined MAD values. This analysis identified *Initial On-site Capacity Fraction* (*IOCF), Limitations Relative to Benefits (LRB), and Total Target Capacity (TTC)* as the three parameters that exert the most influences across both staffing modalities. *LRB* captures the maximum extent to which telehealth limitations can outweigh its benefits, *IOCF* represents the proportion of total workforce capacity initially allocated to on-site staff, while *TTC* represents total staffing capacity across both on-site and telehealth staff (Refer to Supplementary Materials for complete definitions and equations). Thereafter, we conducted univariate testing of these parameters across appropriate value ranges as discussed below, followed by multivariate testing to examine parameter interactions and their effects on outcomes.

To conduct univariate testing of these three parameters, we systematically varied each parameter across a range of values while holding all other parameters constant at their baseline values. For instance, we tested *IOCF* across its full range (0.0–0.9), excluding 1.0 as this represents a system in equilibrium with no telehealth implementation, resulting in no observable changes. We tested selected values for *TTC* (5, 10, 15, 20, 30, 40, 50, and 100) to represent a range of possible facility sizes, deliberately including two extreme values (5 and 100) to test boundary conditions. We tested *LRB* across its full range (0.0–1.0). This approach allowed us to observe how each parameter independently affects system behaviour and identify critical thresholds where small changes in the parameter value produce significant shifts in outcomes.

To examine how these three key parameters interact simultaneously, we conducted multivariate testing using Latin Hypercube Sampling (LHS) for its sampling efficiency, enabling comprehensive exploration of parameter interactions while requiring fewer simulation runs compared to other sampling methods^42–44^. We used uniform distributions to ensure equal probability across each parameter’s range and generated 75 parameter combinations via LHS. Initially, we tested both 75 and 500 samples, but when we observed consistent behaviour and confidence intervals between both sample sizes, we opted to continue with 75 runs as they provided sufficient statistical power while allowing for efficient examination of system behaviours within the parameter space. Our parameter space analysis focused on on-site staff levels as the primary indicator of the system recovery state. To verify our findings were not dependent on a particular sample set, we controlled the randomness across the runs for comparison (See Supplementary Materials).

The parameter range selection for multivariate testing was informed by the results of the univariate testing to capture the full spectrum of system behaviours. For *IOCF*, we selected a range of 0.2–0.9 to maintain a minimum of at least 20% on-site medical capacity for viable healthcare delivery while allowing examination of various telehealth implementation scenarios. For *LRB*, we selected 0.1–1.0, starting at 0.1 rather than 0 to ensure the parameter would meaningfully influence the behaviour of the system. For *TTC*, we selected values between 5 and 50 to represent a range of facility sizes from small clinics to larger ones. All other parameters were set at their baseline values.

### Ethics approval

This research was conducted in accordance with the Declaration of Helsinki and the relevant Australian research guidelines and regulations, including the National Statement on Ethical Conduct in Human Research. To conduct interviews and workshops, we received ethical approval from the Macquarie University Human Research Ethics Committee (REF 520221210042355). All participants provided voluntary informed consent. No compensation was provided.

## Supplementary information

Supplementary information

Supplementary information

## Data Availability

All parameter values and the dataset used for analysis are provided within the manuscript or supplementary materials. The model file can be found here (10.25949/30192943.v3). Interview and workshop transcripts are unavailable due ethical and privacy considerations.
