# Supplementary material for: Conceptualising the impact of telehealth on rural workforce sustainability using system dynamics and the Gartner Hype Cycle
Source: Npj Health Syst. 2025 Oct 29;2:38. doi: 10.1038/s44401-025-00044-1 (PMC13354209; doi:10.1038/s44401-025-00044-1)
Supplement: Supplementary file 1 — Supplementary information [file 44401_2025_44_MOESM1_ESM.pdf]

Conceptualising the Impact of Telehealth on Rural Workforce Sustainability Using System Dynamics and the Gartner Hype Cycle

Sagda Osman*[1], Kate Churruca[a], Mohammad S. Jalali[2], Louise A. Ellis[1], Jeffrey Braithwaite[1]
[1] *Centre for Healthcare Resilience and Implementation Science, Australian Institute of Health Innovation, Macquarie University, North Ryde, Australia.*
[2] *Massachusetts General Hospital Institute for Technology Assessment, Harvard Medical School, Boston, MA, USA*

** Corresponding author*

Email: sagda.osman@mq.edu.au

Variables definitions and equations

This system dynamics model operationalises the Gartner Hype Cycle to examine how clinician-to-clinician telehealth implementation affects rural workforce sustainability through evolving stakeholder perceptions and resource allocation decisions (Refer to the model file here:).

Summary of variables

- **Stocks**: 7
- **Flows**: 9
- **Auxiliaries**: 27
- **Parameters**: 30
- **Total variables**: 73

Stocks

Name	Description	Equation	Units
**On-site staff being hired**	The number of on-site staff currently in the hiring pipeline. This stock accumulates staff who have been hired but have not yet joined the active workforce.	INTEG (On-site staff hiring - On-site staff joining, Initial on-site staff in pipeline)	Person
**On-site staff**	The current number of active on-site staff. This stock tracks current on-site workforce capacity.	INTEG(On-site staff joining - On-site staff departing, Initial on-site staff)	Person
**Telehealth staff being hired**	The number of telehealth staff currently in the hiring pipeline. Follows the same concept as on-site staff being hired, but specific to telehealth roles.	INTEG(Telehealth staff hiring - Telehealth staff joining, Initial telehealth staff in pipeline)	Person
**Telehealth staff**	The current number of active telehealth staff. This stock tracks the current telehealth workforce capacity.	INTEG(Telehealth staff joining - Telehealth staff departing, Initial telehealth staff)	Person

**Perceived telehealth benefits**	Represents the accumulated perception of benefits and value derived from telehealth services among stakeholders. This stock changes based on actual experience and hype/enthusiasm factors.	INTEG(Change in perceived benefits, Initial perceived benefits)	Dimensionless (dmnl)
**Perceived telehealth limitations**	Represents the accumulated perception of limitations and drawbacks of telehealth services among stakeholders. This stock accumulates as evidence of limitations emerges and becomes recognised.	INTEG(Change in perceived limitations, Initial perceived limitations)	Dimensionless (dmnl)
**Perceived Attractiveness of Rural Positions**	The accumulated perception among clinicians regarding the overall appeal and desirability of working in rural on-site medical positions, based clinical support, knowledge transfer opportunities, and medicolegal burden.	INTEG (Change in perceived attractiveness, Initial perceived attractiveness)	Dimensionless (dmnl)

Flows

Name	Description	Equation	Units
**On-site staff hiring**	The rate at which new on-site staff enter the hiring pipeline. This flow is influenced by the on-site staffing gap and constrained by maximum hiring capacity.	MIN (On-site staffing gap / Effective time to hire (on-site), Max on-site hiring)	Person/month
**On-site staff joining**	The rate at which hired on-site staff complete the hiring process and become active.	On-site staff being hired / Average time to join (on-site)	Person/month
**On-site staff departing**	The rate at which on-site staff leave their positions.	On-site staff / Effective time in position (on-site)	Person/month
**Telehealth staff hiring**	The rate at which new telehealth staff enter the hiring pipeline. Similar to on-site staff hiring but specific to telehealth roles.	MIN (Telehealth staffing gap / Average time to hire (telehealth), Max telehealth hiring)	Person/month
**Telehealth staff joining**	The rate at which hired telehealth staff complete the hiring process and become active.	Telehealth staff being hired / Average time to join (telehealth)	Person/month
**Telehealth staff departing**	The rate at which telehealth staff leave their positions.	Telehealth staff / Average time in position (telehealth)	Person/month
**Change in perceived benefits**	The rate at which the perception of telehealth benefits changes over time. Influenced by the gap between hyped benefits and current perceptions.	(Hyped telehealth benefits - Perceived telehealth benefits) / Time to perceive benefits	1/month
**Change in perceived limitations**	The rate at which the perception of telehealth limitations changes over time. Influenced by the gap between	MAX (Limitations activation function*(Evidenced telehealth limitations-	1/month

	evidenced limitations and current perceptions.	Perceived telehealth limitations)/Time to perceive limitations, 0)	
**Change in perceived attractiveness**	The rate at which perceptions of rural positions attractiveness adjust toward indicated levels, influenced by the gap between indicated attractiveness and current perceived attractiveness.	IF THEN ELSE("Initial on-site capacity fraction" = 1, 0, (Indicated attractiveness of rural positions-Perceived attractiveness of rural positions)/Time to adjust perceived attractiveness)	1/month

Auxiliaries

Name	Description	Equation	Units
**Target on-site staff**	The desired number of on-site staff based on total capacity targets and the on-site capacity fraction.	Target total capacity * On-site capacity fraction	Person
**Effective time to hire (on-site)**	The time required to hire on-site staff influenced by the perceived attractiveness of rural on-site positions via the recruitment effect (less attractiveness means longer recruitment timeframes due to limited interested candidates).	Base time to hire (on-site)/Recruitment effect	Month
**Effective time in position (on-site)**	The average duration on-site staff remain in their positions influence by the perceived attractiveness of rural positions via the retention effect (less attractiveness means shorter tenures).	Base time in position (on-site) *Retention effect	Month
**Target telehealth staff**	The desired number of telehealth staff based on total capacity targets and the telehealth capacity fraction.	Target total capacity * (1 - On-site capacity fraction)	Person
**On-site staffing gap**	The difference between target on-site staff and the sum of current on-site staff and staff being hired, adjusted for projected on-site staff loss.	MAX (0, (Target on-site staff - (On-site staff + On-site staff being hired) + Projected on-site staff loss))	Person
**Telehealth staffing gap**	The difference between target telehealth staff and the sum of current telehealth staff and staff being hired, adjusted for projected telehealth staff loss.	MAX (0, (Target telehealth staff - (Telehealth staff + Telehealth staff being hired) + Projected telehealth staff loss))	Person

**Projected on-site staff loss**	Anticipated on-site staff loss based on current attrition rate and the planning horizon.	(On-site staff departing* Hiring planning horizon (on-site)	Person
**Projected telehealth staff loss**	Anticipated telehealth staff loss based on current attrition rate and the planning horizon.	(Telehealth staff departing * Hiring planning horizon (telehealth)	Person
**On-site capacity fraction**	The proportion of total workforce capacity allocated to on-site staff.	SMOOTH (IF THEN ELSE (Initial on-site capacity fraction=1, Initial on-site capacity fraction, MAX (Minimum on-site fraction , (1-Effect of perceived benefits on on-site capacity+ Effect of perceived limitations on on-site capacity*Overreaction multiplier) *Initial on-site capacity fraction)), Capacity adjustment time)	Dimensionless (dmnl)
**Effect of perceived benefits on on-site capacity**	Represents how the perception of telehealth benefits influences the allocation of staff between on-site and telehealth modalities.	Lookup function of Perceived telehealth benefits ([(0,0)-(1,1)],(0,0), (0.1,0.025), (0.2,0.0625), (0.3,0.125), (0.4,0.25), (0.5,0.5), (0.6,0.75), (0.7,0.875), (0.8,0.9375), (0.9,0.975), (1,1), (1.1,1), (1.2,1), (1.3,1), (1.4,1), (1.5,1))	Dimensionless (dmnl)
**Telehealth usage**	The rate at which telehealth services are being used, based on staff numbers and consultation rate.	Telehealth staff * Average telehealth consultations per staff	Consultations /month
**Actual telehealth benefits**	The genuine benefits derived from telehealth based on actual utilisation patterns and outcomes. This variable represents the true value delivered by telehealth, independent of perception or enthusiasm factors. It is calculated using an exponential function to reflect diminishing returns as utilisation increases and to capture how telehealth	(1-EXP(-Telehealth usage/Reference telehealth usage (benefits)))	Dimensionless (dmnl)

	benefits initially grow rapidly with increased usage but gradually level off. The reference usage determines how rapidly these benefits grow with actual usage, calibrating the sensitivity of benefit realisation to usage levels.		
**Hyped telehealth benefits**	The benefits that stakeholders believe telehealth provides, based on actual outcomes and influenced by the hype factor.	Actual telehealth benefits * Hype factor	Dimensionless (dmnl)
**Hype factor**	Represents the hype effect where initial enthusiasm for telehealth is high and then diminishes over time.	1 + (Initial premium * EXP (-Time / Hype decay time))	Dimensionless (dmnl)
**Evidenced telehealth limitations**	The actual limitations of telehealth revealed through utilisation. Calculated using an exponential approach function similar to actual benefits but based on a different reference usage level specific to limitations, signifying that we perceive benefits earlier than we acknowledge limitations.	Limitations relative to benefits*(1-EXP (-Telehealth usage/ Reference telehealth usage (limitations)))	Dimensionless (dmnl)
**Effect of perceived limitations on on-site capacity**	Represents how the perception of telehealth limitations influences the allocation of staff between on-site and telehealth modalities. As limitations of telehealth are recognised, this effect increases the on-site capacity fraction.	Lookup function of Perceived telehealth limitations ([(0,0)-(1,1)],(0,0),(0.1,0.025),(0.2,0.0625),(0.3,0.125),(0.4,0.25),(0.5,0.5),(0.6,0.75),(0.7,0.875),(0.8,0.9375),(0.9,0.975),(1,1))	Dimensionless (dmnl)
**Limitations activation function**	Controls when limitations begin to influence resource allocation decision-making. This function delays the influence of limitations until after the initial enthusiasm/hype period, creating lag/delay between benefits perception and limitations perception	SMOOTH(IF THEN ELSE(Time>Hype decay time , 1 , 0) , Time to perceive limitations)	Dimensionless (dmnl)
**Overreaction multiplier**	A factor that temporarily amplifies the effect of	1 + Overreaction strength * EXP(-(Time - Hype decay	Dimensionless (dmnl)

	perceived telehealth limitations when they are first recognised. This represents the tendency of healthcare organisations to initially overemphasise recognised problems and overreact to them after the initial period of optimism, creating the characteristic "trough of disillusionment" in technology adoption cycles	time)/Overreaction decay time)	
**Overreaction decay time**	The time over which the organisational overreaction to telehealth limitations diminishes. This period could be long if there is an organisational inertia to recalibrate implementation strategies	2*Time to perceive limitations	Month
**Hype decay time**	Time it takes for the initial hype/enthusiasm to decay.	2*Time to perceive limitations	Month
**Telehealth staff ratio**	The proportion of telehealth to total staff, calculated as telehealth staff divided by total staff.	Telehealth staff/(On-site staff + Telehealth staff)	Dimensionless (dmnl)
**Medicolegal burden effect**	Multiplier represents the medicolegal burden on on-site staff due to telehealth tasks that remote clinicians direct but cannot perform themselves. Increasing non-linearly with telehealth staff ratio	Lookup function of telehealth staff ratio ([(0,1)-(1,5)],(0,1), (0.1,1.02), (0.2,1.05), (0.3,1.1), (0.4,1.18), (0.5,1.3), (0.6,1.45), (0.7,1.7), (0.8,1.9), (0.9,2.5), (1,5))	Dimensionless (dmnl)
**Tacit knowledge effect**	Multiplier representing how telehealth staffing affects tacit knowledge transfer and incidental learning for on-site staff due to reduced opportunities for in-person interactions.	Lookup function of telehealth staff ratio ([(0,1)-(1,0)],(0,1), (0.1,1), (0.2,0.98), (0.3,0.92), (0.4,0.8), (0.5,0.65), (0.6,0.5), (0.7,0.35), (0.8,0.2), (0.9,0.1), (1,0))	Dimensionless (dmnl)
**Clinical support effect**	Multiplier representing how the telehealth-to-onsite staff mix affects overall clinical support quality, following an inverted U-shape with optimal support at balanced ratios	Lookup function of telehealth staff ratio ([(0,0)-(1,1)],(0,0.3), (0.1,0.5), (0.2,0.68), (0.3,0.82), (0.4,0.92), (0.5,0.97), (0.6,0.95), (0.7,0.88), (0.8,0.76), (0.9,0.45), (1,0))	Dimensionless (dmnl)

**Indicated attractiveness of rural positions**	The attractiveness level that current telehealth implementation would create if perceptions adjusted instantaneously.	Clinical support effect*Tacit knowledge effect/Medicolegal burden effect	The multiplier represents the medicolegal burden on on-site staff due to telehealth tasks that remote clinicians direct but cannot perform themselves.
**Retention effect**	Multiplier representing how position attractiveness affects staff tenure and turnover rates. Reduced attractiveness results in shorter tenures.	Lookup function of Perceived attractiveness of rural positions ([(0, 0.3)-(1, 1)],(0, 0.3), (0.1, 0.4), (0.2, 0.5), (0.3, 0.6), (0.4, 0.7), (0.5, 0.8), (0.6, 0.9), (0.7, 0.95), (0.8, 0.98), (0.9, 1), (1, 1))	Dimensionless (dmnl)
**Recruitment effect**	Lookup function of Perceived attractiveness of rural positions. Reduced attractiveness results in longer recruitment delays.	([(0, 0.2)-(1, 1)],(0, 0.2), (0.1, 0.25), (0.2, 0.3), (0.3, 0.4), (0.4, 0.5), (0.5, 0.65), (0.6, 0.8), (0.7, 0.9), (0.8, 0.95), (0.9, 1), (1, 1))	Dimensionless (dmnl)

Constants

The values of the constants below represent the base run after pushing the system out of equilibrium by introduction 10% telehealth (i.e., Initial on-site capacity fraction = 0.9).

Name	Description	Value (Base run)	Units
**Initial on-site staff in pipeline**	The starting value for on-site staff in the hiring pipeline.	1.92	Person

**Initial on-site staff**	The starting value for active on-site staff.	9.21	Person
**Initial telehealth staff in pipeline**	The starting value for telehealth staff in the hiring pipeline.	0	Person
**Initial telehealth staff**	The starting value for active telehealth staff.	0	Person
**Initial perceived benefits**	The starting value for perceived telehealth benefits.	0	Dimensionless (dmnl)
**Initial on-site capacity fraction**	The starting allocation of workforce capacity to on-site staff (0.9 means 90% on-site, 10% telehealth). Setting this to values other than 1 pushes the system out of equilibrium.	0.9	Dimensionless (dmnl)
**Base time to hire (on-site)**	The average time required to hire on-site staff.	9	Month
**Average time to join (on-site)**	The average time required for hired on-site staff to become active.	3	Month
**Base time in position (on-site)**	The average duration on-site staff remain in their positions (Average on-site staff tenure).	18	Month
**Average time to hire (telehealth)**	The average time required to hire telehealth staff.	3	Month
**Average time to join (telehealth)**	The average time required for hired telehealth staff to become active.	1	Month
**Average time in position (telehealth)**	The average duration telehealth staff remain in their positions.	6	Month
**Hiring planning horizon (on-site)**	The time horizon used for projecting on-site staff losses.	0	Month
**Target total capacity**	The total desired workforce capacity across both on-site and telehealth modalities.	20	Person
**Hiring planning horizon (telehealth)**	The time horizon used for projecting telehealth staff losses.	0	Month
**Max on-site hiring**	The maximum rate at which new on-site staff can be hired.	5	Person/month

**Max telehealth hiring**	The maximum rate at which new telehealth staff can be hired.	5	Person/month
**Average telehealth consultations per staff**	The average number of consultations a telehealth staff member can deliver per month.	100	Consultations/person/month
**Reference telehealth usage (benefits)**	A calibration parameter representing the benchmark volume of telehealth consultations required to meaningfully evaluate telehealth benefits. This parameter serves as the reference point in the exponential function that calculates actual telehealth benefits based on usage levels.	100	Consultations/month
**Initial premium**	The factor by which actual telehealth benefits are initially inflated due to enthusiasm and optimism at the start of the hype cycle.	0.5	Dimensionless (dmnl)
**Time to perceive benefits**	The average time required to adjust perceptions of telehealth benefits.	3	Month
**Minimum on-site fraction**	The minimum proportion of staff that must be on-site regardless of telehealth benefits.	0.2	Dimensionless (dmnl)
**Initial perceived limitations**	The starting value for perceived telehealth limitations.	0	Dimensionless (dmnl)
**Time to perceive limitations**	The average time required to adjust perceptions of telehealth limitations. Set longer than the time to perceive benefits, reflecting the delayed recognition of limitations.	6	Month
**Reference telehealth usage (limitations)**	A calibration parameter representing the volume of telehealth consultations needed to meaningfully assess limitations. Set higher than the reference usage	300	Consultations/month

	for benefits to signify that limitations often require more extensive use to become apparent.		
**Limitation to benefit ratio**	Defines the maximum potential limitations relative to maximum benefits. Values below 1 ensure that telehealth retains net positive value even when all limitations are fully recognised, preserving incentive for continued utilisation.	0.7	Dimensionless (dmnl)
**Overreaction strength**	Determines the maximum intensity of the initial overreaction to emerging telehealth limitations. Higher values create a deeper trough of disillusionment before the system stabilises.	1.5	Dimensionless (dmnl)
**Initial perceived attractiveness**	Starting value of perceived attractiveness of rural positions	o.5	Dimensionless (dmnl)
**Time to adjust perceived attractiveness**	The average time required to adjust perceptions of telehealth benefits.	3	Month
**Capacity adjustment time**	The time required to adjust capacity allocation in response to changes in perceived benefits and limitations. Represents organisational inertia in resource reallocation decisions.	3	Month

Equilibrium calculations

We provide detailed calculations and justification for the equilibrium parameter values used in our model. Equilibrium represents a stable state where all stocks remain constant over time because inflows equal outflows, and all rates of change equal zero.

In system dynamics models, equilibrium analysis serves multiple purposes including structural validation to ensure the model behaves logically under steady-state conditions, baseline establishment to provide a reference point for dynamic behaviour analysis, and parameter consistency validation to ensure initial values create mathematically consistent

relationships. For workforce models, equilibrium represents a sustainable operating state where recruitment rates balance departure rates, and all perception stocks have adjusted to their indicated values.

At equilibrium, for any stock with inflows and outflows, the rate of change equals zero. Therefore, inflow must equal outflow. For stocks with single flows such as perception stocks, the rate of change equals zero, meaning the stock value must equal its indicated value.

At equilibrium, the model represents a rural healthcare facility that has not yet implemented telehealth services (Initial on-site capacity fraction = 1.0), meaning all clinical capacity is provided through on-site staff. We need to calculate the values for "Initial on-site staff" and "Initial on-site staff being hired" that will maintain this equilibrium state, given all other model parameters are set as specified in Supplementary Material A. Once established, this equilibrium can be disrupted by introducing telehealth capacity (reducing the on-site capacity fraction below 1.0), which will activate the perception dynamics and resource allocation changes that form the core of our model.

Equilibrium Calculation

Other parameters values

Target total capacity = 20 person

Initial on-site capacity fraction = 1.0 dimensionless [for equilibrium]

Initial perceived attractiveness = 0.5 dimensionless

Base time to hire (on-site) = 9 month

Average time to join (on-site) = 3 month

Base time in position (on-site) = 18 month

Hiring planning horizon (on-site) = 0 month

Max on-site hiring = 5 person/month

Calculating Recruitment and Retention Effects at Attractiveness = 0.5

The model assumes rural positions start with moderate attractiveness (0.5 on a scale of 0-1), reflecting the inherent challenges of rural healthcare practice. This attractiveness level affects both recruitment difficulty and staff retention through lookup functions defined in the model.

From the lookup functions in the document:

Recruitment effect = 0.65 dimensionless [from lookup function at attractiveness = 0.5]

Retention effect = 0.8 dimensionless [from lookup function at attractiveness = 0.5]

Calculating Effective Recruitment and Retention Timeframes

The workforce effects directly influence recruitment and retention timeframes. Reduced attractiveness makes positions harder to fill (longer recruitment times) and increases turnover (shorter tenure).

Effective time to hire (on-site) = Base time to hire (on-site) / Recruitment effect

Effective time to hire (on-site) = 9 / 0.65 = 13.846 month

Effective time in position (on-site) = Base time in position (on-site) × Retention effect

Effective time in position (on-site) = 18 × 0.8 = 14.4 month

Setting Up Equilibrium Conditions

For the system to be in equilibrium, the rate at which staff join the active workforce must equal the rate at which they depart. Additionally, the rate at which new staff are hired into the pipeline must equal the rate at which they complete training and join the active workforce.

On-site staff joining = On-site staff departing

On-site staff being hired / Average time to join (on-site) = On-site staff / Effective time in position (on-site)

On-site staff being hired / 3 = On-site staff / 14.4

Therefore:

On-site staff being hired = On-site staff × (3 / 14.4) = On-site staff × 0.208

Calculating Staffing Gap (with planning horizon = 0)

The staffing gap represents the difference between desired staffing levels and current capacity (including staff in the pipeline). With planning horizons set to zero, the model does not account for projected future losses when calculating hiring needs, simplifying the equilibrium calculation (and reflecting reality where hiring is mostly reactive!).

Target on-site staff = Target total capacity × Initial on-site capacity fraction

Target on-site staff = 20 × 1.0 = 20 person

Projected on-site staff loss = 0 person [planning horizon = 0]

On-site staffing gap = MAX(0, Target on-site staff - (On-site staff + On-site staff being hired))

On-site staffing gap = MAX(0, 20 - (On-site staff + On-site staff × 0.208))

On-site staffing gap = MAX(0, 20 - On-site staff × 1.208)

Calculating Hiring Rates

The actual hiring rate is constrained by both the staffing gap (demand for staff) and the maximum hiring capacity (organisational constraint). At equilibrium, this hiring rate must equal the departure rate to maintain stable stock levels.

Desired hiring rate = On-site staffing gap / Effective time to hire (on-site)

Desired hiring rate = (20 - On-site staff × 1.208) / 13.846

Actual hiring rate = MIN(Desired hiring rate, Max on-site hiring)

Actual hiring rate = MIN((20 - On-site staff × 1.208) / 13.846, 5)

At equilibrium:

Actual hiring rate = On-site staff joining = On-site staff / 14.4

Solving for Equilibrium

To find the staffing level where hiring demand exactly matches hiring response, creating a stable equilibrium state:

If not constrained by maximum hiring:

(20 - On-site staff × 1.208) / 13.846 = On-site staff / 14.4

(20 - On-site staff × 1.208) × 14.4 = On-site staff × 13.846

288 - On-site staff × 17.395 = On-site staff × 13.846

288 = On-site staff × (17.395 + 13.846)

288 = On-site staff × 31.241

On-site staff = 288 / 31.241 = 9.218 person

On-site staff being hired = On-site staff × 0.208 = 9.218 × 0.208 = 1.917 person

Model testing

We conducted sensitivity analysis by varying each parameter by ±10% from its base value and measuring its impact using mean absolute deviation (MAD). We ran these tests in Vensim PLE Plus using the Sensitivty2All function in Vensim PLP (Figure S1 and Figure S2). We calculated the average MAD values (for MAD corresponding to the ±10% change in parameter value) for the top most influential parameters.

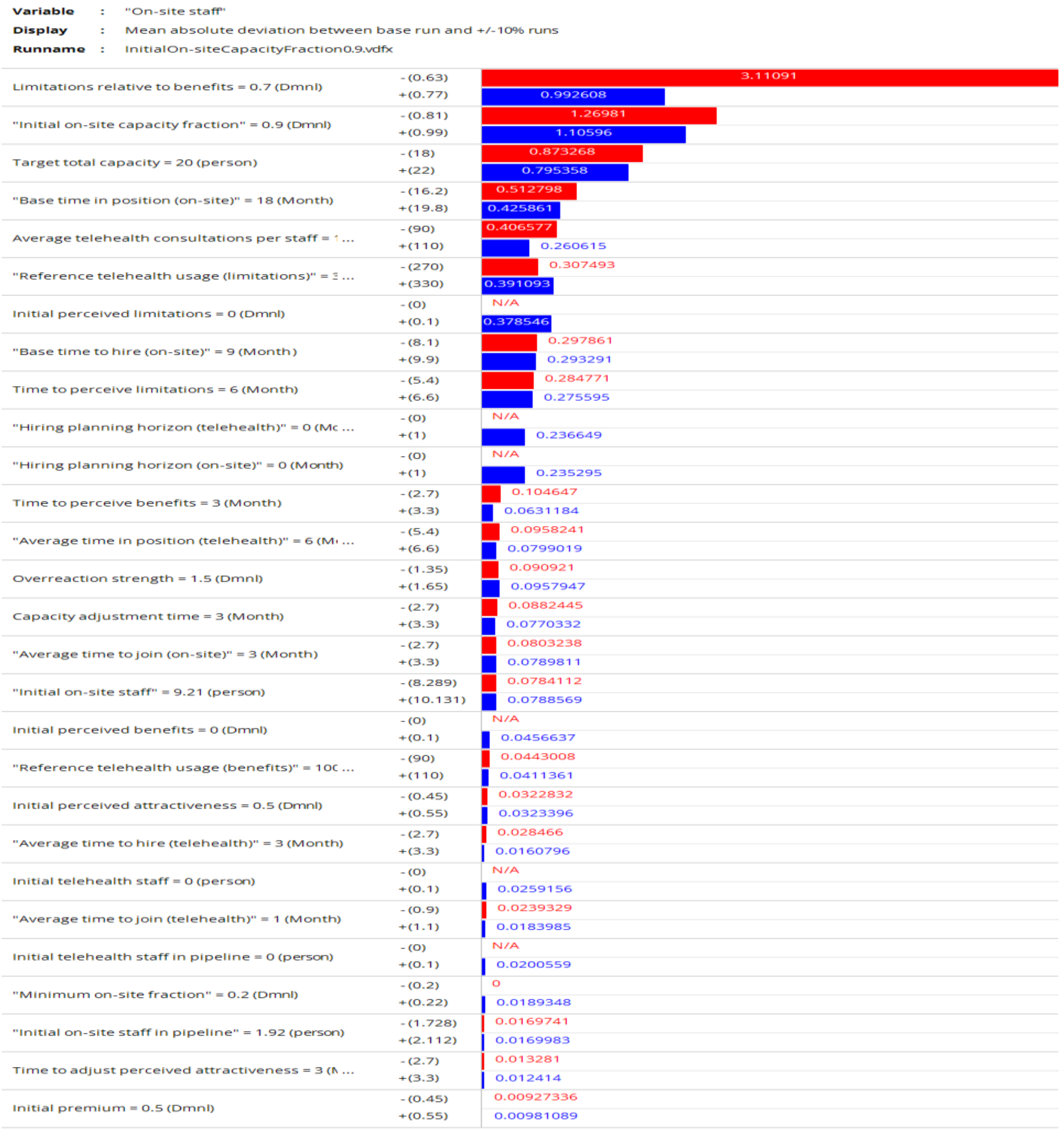

Figure S1: Results of sensitivity testing (Sensisitvty2All function in Vensim PLP) for on-site staff using mean absolute deviation between base run and +/-10%.

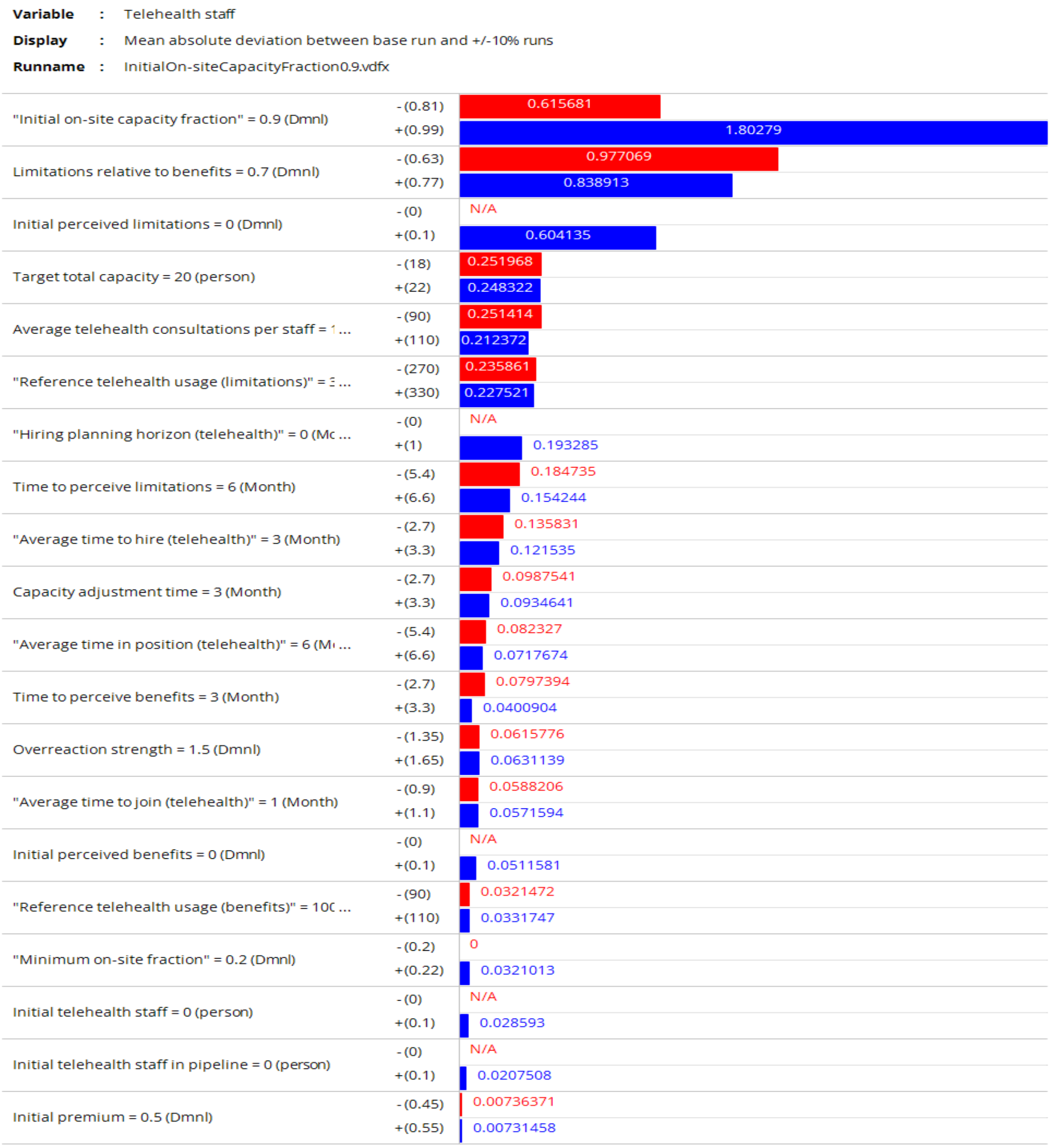

Figure S2: Results of sensitivity testing (Sensisitvty2All function in Vensim PLP) for on-site staff using mean absolute deviation between base run and +/-10%.

Through this analysis, we identified three parameters that exerted the strongest influence on the system behaviour:

1. *Limitations relative to benefits (LRB)*: This parameter (set at 0.7 in the base run) captures the maximum extent to which telehealth limitations can outweigh its benefits in a specific setting, reflecting organisational readiness and the ceiling of stakeholders' perceptions about telehealth's limitations compared to its perceived value. Lower values indicate limitations have less potential to detract from benefits,

while higher values mean perceived limitations can substantially undermine perceived benefits. It was the most influential for on-site staff (average MAD: 2.05) and second most influential for telehealth staff (average MAD: 0.91).

2. *Initial on-site capacity fraction (IOCF):* This parameter (set at 0.9 in the base run) represents the proportion of total workforce capacity initially allocated to on-site staff versus telehealth. It dominated sensitivity for telehealth staff (average MAD: 1.21) and ranked second for on-site staff (average MAD: 1.18).

3. *Target total capacity (TTC):* This parameter (set at 20 in the base run) represents the total staffing capacity of the facility combining both on-site and telehealth staff, reflecting the facility size. It showed high influence on on-site staff (MAD: 0.83) and moderate influence for telehealth staff (MAD: 0.25).

A noteworthy observation was the pronounced asymmetry in parameter responses. For instance, increasing *IOCF* by 10% had a dramatically larger effect on telehealth staff levels (MAD: 1.8) than decreasing it by 10% (MAD: 0.62). Similarly, decreasing *LRB* had a substantially stronger effect on on-site staff (3.11) than increasing it (0.99). This asymmetry suggests non-linearities in the system's response, indicating critical thresholds where small adjustments can trigger disproportionate shifts in system behaviour.

To conduct multivariate testing, we initially tested both 75 and 500 samples, but when we observed consistent behaviour and confidence intervals between both sample sizes, we opted to continue with 75 runs as they provided sufficient statistical power while allowing for further examination of system behaviours within the parameter space. We used a random seed of 1234 but also repeated the testing with a different seed (456) and observed consistent results, confirming that our findings reflect the system behaviour rather than anomalies due to a particular sample set. To understand the distribution of system behaviours within the parameter space, we classified these runs into two categories based on their behaviour following the trough of disillusionment (Refer to Supplementary Data S1).
